# An exploratory study of fluid and carbohydrate intake and related metabolic and hormonal responses during intermittent mixed racewalking: implications for the Olympic mixed racewalking relay

**DOI:** 10.3389/fnut.2026.1798548

**Published:** 2026-07-03

**Authors:** Francisco Javier Martínez-Noguera, Pedro E. Alcaraz, Josu Gómez-Eceiza, Cristian Marín-Pagán

**Affiliations:** 1 Research Center for High Performance Sport, Catholic University of Murcia, Murcia, Spain; 2 Royal Spanish Athletics Federation (RFEA), Madrid, Spain

**Keywords:** adrenaline, carbohydrates, glucagon, glucose, insulin, noradrenaline, recovery, subsequent exercise

## Abstract

The new racewalking event at the 2024 Paris Olympics was a mixed marathon relay. This poses new physiological challenges, as this competition format has never been attempted before. Therefore, this study will help generate new insights into how metabolism and the endocrine system function and interact in this novel format. This would allow for the identification of limitations and adjustments in the future for this type of protocol. A pilot study was conducted to characterize glycemic and hormonal responses–including glucose (GLUv), insulin (IN), adrenaline (A), noradrenaline (NA), glucagon (GG), and cortisol (C) in plasma during a structured protocol designes mimic the event. The protocol consisted of two high-intensity exercises with an interspersed rest period (40 min of exercise at 90% of HRmax + 40 min of active recovery + 40 min of exercise at 90% of HRmax), conducted under a predefined fluid and carbohydrates (CH) intake strategy. During this protocol, subjects ingested 50 g CH in 500 ml in each phase of the protocol and, in addition, a 45 g CH gel during recovery. No hypoglycemia or dehydration >2% of body mass (BM) was observed throughout protocol. In addition, IN (↑~84%) and GLUv (↑~41%) evolved similarly in both exercise sectors. Despite the carbohydrate and fluid intake strategy, increases in counterregulatory hormones, particularly in NA (161%) and GG (42%), were observed during the second phase of exercise. Within the limitations of this exploratory study, these findings describe the hormonal and metabolic response patterns observed under the conditions tested, without implying causal or functional effects of the intake strategy. The high interindividual variability and small sample size prevent any statistical inferences from being made. In addition, high carbohydrate intake during recovery was accompanied by an increase in IN concentration, with no evidence of hypoglycemia before the second exercise segment.

## 1 Introduction

In 2024, the racewalking competition in the mixed marathon relay was introduced, which was the first time it was included at the Olympic Games in Paris 2024. Previously, in the Olympic Games the 20 km and 50 km racewalking events were held, but in Paris 2024 they substituted the 50 km with a mixed relay. In this new competition, a man and a woman were required to run a 42 km relay in the following sequence: 1) man 11.4 km, 2) woman 10.0 km, 3) man 10.0 km and 4) woman 10.8 km. This new competition format means that the athletes must wait until their second relay for about 40 min. This poses a meaningful physiological challenge, as the 40-min period requires optimal planning to modulate core and peripheral temperature, replenish fluids and energy, and manage metabolism and the neuromuscular system in order to begin the second part of the exercise in peak performance condition.

The different sectors of the mixed racewalking relay were performed at high intensity around of approximately 85–95% of maximal oxygen consumption (VO_2_MAX__), which is above the respiratory compensation point (RCP o VT2) for most athletes, and consequently where the predominant metabolic fuel is carbohydrates (1, 2). Glucose regulation during exercise is tightly controlled by precise physiological mechanisms to maintain plasma glucose (GLUv) within a narrow range that ensures adequate energy supply, particularly for the brain (3). Normal GLUv levels are ~100 mg/dl, whereas progressive reductions GLUv suppress insulin (IN) secretion and stimulate counter-regulatory hormones such glucagon (GG), adrenaline (AD), growth hormone (GH) and cortisol (COR) at defined thresholds (4–6). During high intense exercise, rapid muscle glycogen utilization and competition between skeletal muscle and brain for GLUv increase the risk of hypoglycemia (3). The balance between hepatic glucose production and peripheral glucose uptake is dynamically regulated by endocrine responses, particularly the glucagon-to-insulin ratio and sympathetic activation, which increase hepatic glycogenolysis and gluconeogenesis (7). When GLUv falls below critical levels, cerebral metabolism and cognitive function are impaired, potentially leading to severe consequences (4–6).

During prolonged submaximal exercise, muscular GLUv increase to meet energy demands, while hepatic glucose release temporarily rises to help maintain GLUv levels (8). Although a gradual decline in GLUv levels is observed, it typically does exceed 10–15% due to compensatory hepatic glucose production and hormonal regulation (8–10). If exercise follows, endocrine responses adjust to stabilize GLUv concentrations. This control is mediated by the sympathetic nervous system, which modulates pancreatic secretion by reducing IN and increasing GG via activation of α- and β-adrenergic receptors, respectively (11).

The plasma GG/IN ratio directly affects hormonal control during prolonged submaximal exercise, while nervous system factors drive immediate increase in neoglycogenic activities and hepatic glucose release (12–15). In prolonged exercise, GG production is mainly regulated by decrease GLUv, which stimulates hepatic glucose production but not muscle cells (16). GG also stimulates lipolysis in adipocytes, raising plasma free fatty acids (FFA) for used in the muscle cell and other tissues, and increasing plasma glycerol for hepatic neoglycogenesis. Ketone bodies and FFA can further provide negative feedback on GG synthesis (17).

One of the adaptations to endurance training that has been seen is that, during prolonged submaximal exercise, the decrease in IN and increase in GG are attenuated (18). It seems that trained athletes can maintain optimal GLUv levels with less fluctuations in the mentioned hormones and this is due to a lower activation of the sympathetic system mediated by a lower plasma circulation of noradrenaline (NA) and A (catecholamines) for the same intensity (18). These inhibit the production of IN and increase plasma concentrations of GG, to maintain optimal GLUv levels (18).

AD and NA act on the liver to increase neoglycogenesis and glycogenolysis, while also stimulating glycogenolysis in skeletal muscle and lipolysis in fatty tissue (19–21). Catecholamines are released as NA (~95%) and A (~5%) at postganglionic sympathetic endings, but the adrenal medulla release ~20% NA and ~80% E (22). During exercise above 50–60% of the VO_2_MAX__, NA and AD effects are minimal, and the GG/IN ratio become the main regulator of GLUv (23). Prolonged submaximal exercise-induced catecholamine increases also support circulatory demands for thermoregulation, where higher body temperature triggers cutaneous vasodilation, reduced venous and right ventricular filling pressures, decreased stroke volume, and compensatory increases in heart rate and splanchnic vascular resistance via NA to maintain cardiac output (24).

COR secretion increases during exercise, rising with intensity and showing a critical threshold at 50–60% of VO_2_MAX__, which can shift upward shift training, resulting in lower COR responses at the same intensity (25–28). During submaximal exercise below this threshold, plasma COR may remain at or below resting levels, but prolonged low intensity exercise can elevate it, above the threshold, COR initially rises and then stabilizes, with duration and intensity influencing this plateau (25–27, 29). Environmental temperature and low-carbohydrate diets can further modulate COR levels during submaximal exercise (26, 30, 31). COR maintains euglycemia via hepatic gluconeogenesis, enzyme expression, amino acids mobilization, inhibition of GLUv into muscle cells and adipocytes, and stimulation of lipolysis in adipocytes; it also facilitates the conversion of NA into AD (32, 33). Lack of COR response can reduce catecholamine levels and compromise exercise performance, while catecholamines in turn can suppress COR secretion (34, 35).

As was previously observed, glycemic control during prolonged submaximal exercise involves complex hormonal regulation to avoid hypoglycemia. But following exercise, a series of metabolic and hormonal changes occur with the aim of readjusting GLUv coupled to an increase in muscle glycogen resynthesis even without exogenous carbohydrate (CH) intake (36). However, after CH ingestion post-exercise, muscle glycogen resynthesis is higher compared to without CH ingestion (37, 38), improving performance in a subsequent prolonged submaximal exercise compared to placebo (39). The rate of glycogen resynthesis is maximized by consuming carbohydrates right after exercise, but it can be lowered to about 45–50% if it is delayed by even 2 h (38). It has been demonstrated that quantities near 1.2 g CHO/kg/h during the early post-exercise phase are ideal for glycogen resynthesis when recovery time is restricted (< 8 h) (40). In this regard, several studies have shown how CH intake (50–382 g for 4 h of recovery) after prolonged submaximal exercise improved performance in a subsequent endurance test (41–43). The frequent intake of large amounts of CH during recovery allows the establishment of high GLUv and IN levels which leads to a decrease in FFA concentrations. As a consequence, a decrease in fat oxidation can be observed during subsequent prolonged submaximal exercise (39, 43). Additionally, glycogen resynthesis during post-exercise recovery can be adversely affected by dehydration and hyperthermia, indicating that maintaining proper hydration may foster a more favorable metabolic environment for glycogen replacement (44).

However, despite extensive research on CHO metabolism during continuous exercise, most studies have examined either single exercise bouts or long recovery periods (>3–4 h), which do not reflect the short recovery intervals characteristic for modern competition formats such as relay endurance events (45). Furthermore, previous investigations have typically evaluated only isolated hormonal responses rather than simultaneously assessing the integrated endocrine network regulating glycemia, including IN, GG, A, NA, COR, or dopamine (DP) (36, 39, 41–43). This lack of information makes it difficult to understand the mechanisms regulating glycemia restoration in competition-realistic contexts.

Critically, there is a lack of studies investigating the integrated metabolic and hormonal responses during repeated high-intensity exercise bouts separated by short recovery periods with structured carbohydrate intake. This represents a major gap in current sports physiology literature, particularly given the increasing prevalence of intermittent endurance competition formats (46). Understanding how CHO intake strategies influence glycemic stability and endocrine regulation under these conditions is essential to optimize athlete safety, embolic stability, and performance.

The mixed racewalking relay event held at the Paris 2024 Olympic Games presents a very different effort pattern: ~40 min of exercise + ~40 min of recovery + ~40 min of exercise. This structure demands rapid hormonal adjustments and the efficient availability of GLUv. Athletes must maintain glycemic homeostasis within short time intervals to prevent hypoglycemia, dehydration, and performance decline—factors historically associated with increased dropout risk and reduced competitive capacity.

To date, no study has simultaneously examined the integrated hormonal and glycemic responses during a repeated exercise-recovery-exercise protocol of this duration under controlled CHO intake conditions. Addressing this gap is essential to improve understanding of metabolic regulation, optimize CHO intake strategies, and inform evidence-based guidelines for athletes performing repeated endurance efforts with limited recovery time.

Given that, to our knowledge, no previous study has simultaneously evaluated a complete set of glucose-regulating hormones within an exercise-recovery-exercise model that replicates the temporal structure of the Olympic mixed racewalking relay (40 min of exercise, 40 min of recovery, and 40 min of exercise). This pilot and feasibility study aims to descriptively explore preliminary patterns of hormonal and metabolic responses associated with GLUv maintenance within a protocol characterized by two high-intensity exercise bouts separated by a relatively short recovery period. Rather than claiming mechanistic novelty, the study focuses on the integrated assessment of multiple glucose-regulating hormones within this specific temporal structure, which has been insufficiently characterized in previous literature. In addition, the study seeks to identify potential physiological risks (hypoglycemia during recovery and dehydration) and assess the logistical and physiological feasibility of the protocol, including total duration, tolerance to CH intake, and ability to complete both exercise sessions.

In this context, the pilot study will assess whether the protocol is safe, operationally feasible, and scientifically sound, providing essential data on recruitment rates, completion, tolerance to CH intake, hormonal variability, and glycemic responses. These preliminary outcomes will help refine workload parameters, sampling timings, and ensure that a full-scale study can be conducted with methodological and ethical rigor.

It is hypothesized that, in a protocol simulating the mixed relay race walking format (40 min of exercise −40 min of recovery −40 min of exercise), this pilot study explores whether a structured carbohydrate and fluid intake strategy is feasible and well tolerated, enabling athletes to complete both exercise bouts without clinically relevant hypoglycemia or excessive dehydration. Furthermore, it is hypothesized that glycemic homeostasis will be maintained through coordinated hormonal response patterns, characterized by parallel changes in GLUv and IN concentrations and by increased activation of counterregulatory hormones (e.g., glucagon and catecholamines), specifically in the second exercise segment. Given the exploratory nature of this pilot study, these hormonal and metabolic responses were expected to show interindividual variability and serve primarily as preliminary physiological trends to inform the design of future controlled trials.

## 2 Methods

### 2.1 Participants

Five endurance trained runners were recruited to perform this study with the following characteristics: mean (standard deviation) age 38.7 ± 4.03 kg, height 176.7 ± 5.31 cm, weight 70.0 ± 5.43 kg, body mass index (BMI) of 22.3 kg·m^−2^, % fat mass 9.6 ± 1.10 % and % muscle mass 51.3 ± 0.56 %. Inclusion criteria were: 18–40 years of age, BMI of 19.0–25.5 kg·m^−2^, 6–12 h minimum of training per week (at least 4 sessions per week) and 3 years of trail running experience. However, subjects were excluded if they had metabolic health problems, were smokers or regular alcohol consumers, had suffered an injury in the last 6 months, had cardiorespiratory or digestive disease, had taken any supplement or medication in the 2 weeks prior to the start of the study, and had abnormal values in the health blood test. Due to practical constraints in recruiting competitive race walkers capable of completing the full experimental protocol, participants were selected from a trained running population, allowing control of internal load while acknowledging the limitation in direct sport-specific transferability. Participants who were included in the study gave informed consent prior to the start date of the investigation. This research was in accordance with the guidelines of the Declaration of Helsinki Declaration on Human Research (47) and was approved by the Ethics Committee of the university (CE012104). All participants successfully completed the study.

### 2.2 Study design

This investigation used an observational pilot and feasibility design. Participants were well-trained runners who completed an exercise protocol that included a 40-min rectangular test at an intensity of 90% of maximal heart rate (HR_MAX_), followed by a 40-min recovery phase (an active part) and a second 40-min rectangular test at the same relative intensity. No experimental manipulation or comparator condition was applied.

A comprehensive set of hormonal biomarkers were examined: IN, GC, AD, NA, COR and DP, metabolic: plasma and capillary glucose (GLUv and GLUc), triglycerides (TG), free fatty acids (FFA), lactate (LAC) and resting heart rate (HR) throughout the exercise protocol. Given the reduced sample size and the specificity physiological model employed, this study was designed as a descriptive feasibility investigation aimed characterizing response patterns, assessing protocol tolerance and logistical viability, and generating preliminary data to inform the insights of future controlled studies, rather than providing confirmatory or causal evidence.

### 2.3 Procedures

**Figure 1:**
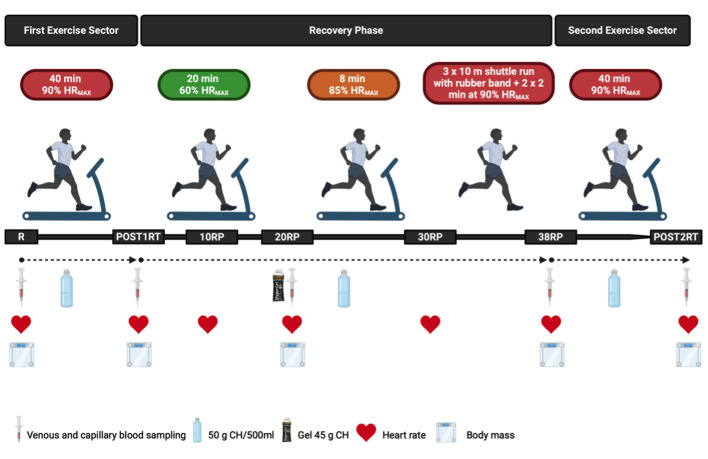
Heart rate (HR) kinetics during the exercise protocol. Data are presented individually.

The subjects included in the study visited the laboratory three times. On the first visit, the participants were informed of the procedures and tests that would be performed during the study, and they also underwent a medical examination to check their health status. Body composition was evaluated by anthropometry and the participants were informed that they should follow an established diet (by a nutritionist) the day before performing the exercise protocol and that they should not train in the 24 h prior. At this first visit, familiarization of the exercise protocol was performed (1st rectangular test (40 min) + recovery phase 40 min + 2nd rectangular test). In the second visit, a maximal incremental test was performed to know the HR_MAX_ of each subject. In the third visit, the exercise protocol was performed and during these 5 extractions of venous and capillary blood were performed to analyze the biomarkers mentioned above. During the recovery phase, the subjects performed the first 20 min at 60% HR_MAX_ to accelerate LAC reduction without losing much temperature at the muscle level, then performed 8 min at 85% HRmax to activate metabolism and raise muscle temperature, ending with 2 min at 90% HRmax for neuromuscular activation (Figure 1).

On the day before visit 3, runners consumed a standardized diet containing 0.93 g/BM fat, 1.48 g/BM protein, and 9.10 g/BM carbohydrate, also ingested 2.0–2.5 L/day of water. In addition, subjects were required to eat a standardized breakfast 2 h before testing, consisting of 0.45 g/BM protein, 1.32 g/BM carbohydrate, and 0.60 g/BM fat. In addition, an intake of 7 ml/kg of body weight of water 2 h before exercise. The previous day's meals and pre-test breakfasts were prepared by a sports nutritionist and sent to the participants 1 week before the start of the study.

### 2.4 Tests

#### 2.4.1 Health examination

The health assessment involved evaluating the participant's medical history, a resting electrocardiogram, and a medical examination by a doctor including auscultation and blood pressure measurement to confirm the participant's suitability for the study based on their health.

#### 2.4.2 Health blood sample

A certified nurse conducted the venous blood extraction, acquiring one 3 ml tube of ethylenediaminetetraacetic acid (EDTA) for hemogram analysis and another 3.5 ml tube with polyethylene terephthalate (PET) for health assessment. An automated Cell-Dyn 3700analyzer (Abbott Diagnostics, Chicago, IL, USA) was used to conduct a red blood cell count, with internal (Cell-Dyn 22) and external (Program of Excellence for Medical Laboratories-PEML) controls being utilized. The analyzer estimated values of erythrocytes, hemoglobin, hematocrit and hematimetric indexes.

#### 2.4.3 Anthropometry

An anthropometric study was carried out by a researcher certified by the International Society for the Advancement of Kinanthropometry (ISAK) Level 1. Height and body weight were measured using a clinical digital scale with a stadiometer (SECA 780; Vogel & Halke GmbH& Co., Hamburg, Germany). Skinfold thickness was measured using Holtain Skinfold Calipers (Holtain, Ltd., Crymych Pembrokeshire, UK) following the ISAK guidelines (48). Fat percentage was established with the Faulkner formula (49), while muscle mass percentage was computed with the adapted Matiegka equation (50). Also calculated was the sum of the eight skinfolds: triceps, subscapular, bicep, iliac crest, supraspinal, abdominal, thigh and calf.

#### 2.4.4 Maximal incremental test

A progressive maximal test was conducted on a treadmill (Trackmaster treadmill, Newton, KS, USA) (1,029 mBar). The assessment began with a 5-min warm up at a speed of 5.0 km/h, followed by a stage at 9.0 km/h lasting 2 min, after which speed was increased by 1.0 km/h every 2 min until reaching either technical or physiological exhaustion. The information from this test was utilized to define the HR_MAX_ for the exercise protocol.

#### 2.4.5 Familiarization and exercise protocol investigated

The exercise protocol tested in our study was composed of a first rectangular test (1RT) of 40 min of constant work at 90% of HR_MAX_, followed by a recovery phase (RP) consisting of 20 min at 60% HR_MAX_ (20RP) + 8 min 85% HR_MAX_ + 3 x 10 m shuttle run with rubber bands + 2 x sets of 2 min at 90% HR_MAX_ (38RP) and ending with a second RT (2RT) of 40 min of constant work at 90% of HR_MAX_ (Figure 1). The RP was deliberately designed as a structured phase of active recovery and transition, rather than a period of passive recovery, in order to better replicate the actual competitive context of the mixed-race walking relay. In this event, athletes typically remain physically active between sets, performing low-intensity movements, preparatory exercises, and brief changes in intensity before the second effort. Therefore, the inclusion of moderate- and high-intensity segments within the RP was intended to simulate these physiological and neuromuscular demands, while allowing for partial metabolic recovery. The RP should be interpreted as a hybrid recovery/transition phase: although it includes short high-intensity series, most of the period is performed at 60% of HR_MAX_, allowing for partial metabolic recovery between the two rectangular tests (1RT and 2RT). The entire exercise protocol was performed on a treadmill (Trackmaster treadmill, Newton, KS, USA), except for the shuttle run with rubber band. Before starting the exercise protocol, a 15 min warm-up at 60% of HR_MAX_ was performed. In addition, we registered the HR at rest (R), after 1RT (POST1RT), at 10 min RP (10RP), at 20 min RP (20RP), at 30 min RP (30RP), at 38 min RP (38RP) and after the end of 2RT (POST2RT). During 1RT, RP and 2RT the trained runners took a CH and sodium-containing beverage, in addition, 20 min into the RP they ingested a gel containing CH and sodium. In addition, a stop for blood sampling was performed after 1RT, at 20 min PR and at 38 min PR. In the present study, exercise intensity was prescribed and monitored using relative heart rate (%HR_MAX_) as a practical substitute for internal load. Although %HR_MAX_ is commonly used in field-based protocols, it should be noted that heart rate does not provide a direct measure of metabolic intensity and may be influenced by factors such as cardiovascular drift, hydration status, and interindividual variability (51). For this reason, glycogen stores can be depleted during exercise lasting between 45 and 90 min at moderate-high intensity (52, 53). This can lead to the onset of fatigue which is related to the depletion of endogenous carbohydrate (glycogen) stores (54). The temperature at which the tests were performed in the laboratory was 23.1–24.5°°C and a relative humidity of 48–53%. All exercise trials were conducted at the same time of day to minimize circadian influences on hormones.

#### 2.4.6 CH intake strategy during the exercise protocol

During the 1RT, RP and 2RT, participants ingested a drink distributed in small aliquots every 8 min, corresponding to a total intake of 500 ml per phase (1RT, RP and 2RT), providing 50 g CH and 112 mg sodium (Hyperdrink 90, Crown Sport Nutrition) per phase, in water at a temperature of 12–15°°C. This corresponds to an approximate intake rate of ~75 g CH·h^−1^, which is consistent with current recommendations for prolonged high-intensity endurance exercise using multiple transportable carbohydrates. In addition, 20 min into the PR the runners took a gel with 45 g CH and 165 mg sodium (Hypergel 45, Crown Sport Nutrition). Total carbohydrate intake throughout the protocol was ~195 g, within physiologically tolerable ranges and without exceeding recommended oxidation capacities when combining maltodextrin and fructose. HyperDrink 90 and Hypergel 45 contain a mixture of maltodextrin and fructose, in a 1:0.8 ratio. The intake of the CH drink and the gel produced no gastrointestinal problems in the participants.

#### 2.4.7 Blood sampling during the exercise protocol

During the exercise protocol, two types of blood samples were drawn, one venous and one capillary. Five extractions of both types of samples were performed: at R, after POST1RT, at 20RP, at 38RP and POST2RT. Venous blood samples were used to analyze hormonal biomarkers IN, GC, AD, NA, COR and DP, and metabolic biomarkers GLUv, TG and FFA. Finger capillary blood samples were used to analyze GLUc and LAC.

Venous blood samples were collected in R, POST1RT, 20RP, 38RP and POST2RT, centrifuged at 3,000 rpm at 4 °C for 10 min, separating plasma from cells. Glucose was determined using the glucose oxidation method (glucose oxidase/peroxidase) and triglycerides by the triglyceride oxidation method (glycerol phosphate oxidase/peroxidase) using the Biosystems measurement kit (Biosystems S.A., Barcelona, Spain) and according to their instruction manuals. Plasma aliquots were temporarily stored at −20 °C.

IN and COR plasma were analyzed by electrochemiluminescence immunoassay with the Elecsys Insulin and Elecsys Cortisol II measurement kit (Cobas, Roche Diagnostics GmbH, IN, USA), according to their instruction manuals. Measurement of free fatty acid concentration was performed with an enzymatic colorimetric method, using the Wako NEFA C kit (Wako Chemicals, Inc., Richmond, VA, USA) according to the manufacturer's instructions. Plasma GG concentrations were analyzed by ELISA kit (10-1271-01) (Mercodia AB, Uppsala, Sweden) with an intraassay coefficient of variation (CV) of 2%.

Catecholamine concentrations were analyzed by high-performance liquid chromatography with electrochemical detection (Chromsystems^Ⓡ^, Munich, Germany). Sensitivity and intra- and intraassay coefficients of variation were, respectively, 2.3% and 5.2% for DP, 2.9% and 6.9% for NA, 4.3% and 8.5% for AD.

#### 2.4.8 Measurement of biomarkers in capillary blood

The measurements collected prior to, during, and after the exercise protocol were examined using a Lactate Pro2 device (LT-1,730; Arkray, Japan) on blood samples taken from the fingers. An Accu-Chek Performa (Roche, Switzerland) was used for finger glucose measurement (GLUc). The specific test strips for this glucose meter use a mutant variant of the glucose dehydrogenase quinoprotein (Mut. Q-GDH). For capillary blood sampling, 23G (gauge) lancets with 2.0 mm penetration depth manufactured by MenaLancetPro (Menarini Diagnostics, Barcelona, Spain) were used.

#### 2.4.9 Statistical analysis

IBM Social Sciences software (SPSS, v.21.0, Chicago, IL, USA) was used for statistical analysis. Given the pilot and exploratory nature of the study and the small sample size, the data were analyzed primarily using descriptive statistics, prioritizing the magnitude, direction, and variability of changes throughout the protocol. Results are presented as mean ± standard deviation (SD), together with 95% confidence intervals (95% CI) and the coefficient of variation (CV) as an indicator of interindividual dispersion. The coefficient of variation was used to evaluate the homogeneity or heterogeneity of physiological and metabolic responses among participants at each time point, allowing for a more detailed interpretation of the consistency of responses across the different phases of the protocol (exercise–recovery–exercise).

Additionally, the area under the curve (AUC) for GLUc and GLUv was calculated across the entire protocol using the trapezoidal method, in order to integrate the overall glycemic response in both compartments. Differences between GLUc and GLUv AUC were explored using paired AUC comparisons, solely to characterize the magnitude and direction of potential systematic discrepancies between the two measurement methods, without inferential intent. Given the nature of the study, no extensive inferential analyses or multiple comparisons were performed, thereby avoiding interpretations based solely on statistical significance. Instead, the analysis focused on the physiological interpretation of the observed changes, considering the temporal evolution of the variables, the coincidence or displacement of peak responses between compartments, and interindividual variability.

This approach is consistent with methodological recommendations for pilot studies, whose primary objectives are to assess protocol feasibility, evaluate the sensitivity of the measured variables, and generate hypotheses for future studies with greater statistical power (55).

## 3 Results

This pilot feasibility study included five participants and aimed to characterize the metabolic, hormonal, autonomic, and perceptual responses to an exercise protocol consisting of an initial exercise bout (R-POST1RT), an intermediate recovery period (POST1RT-38RP), and a second exercise bout (38RP-POST2RT).

### 3.1 Glycemic and insulinemic variables

#### 3.1.1 Comparison between capillary and venous glycemia

**Figure 2:**
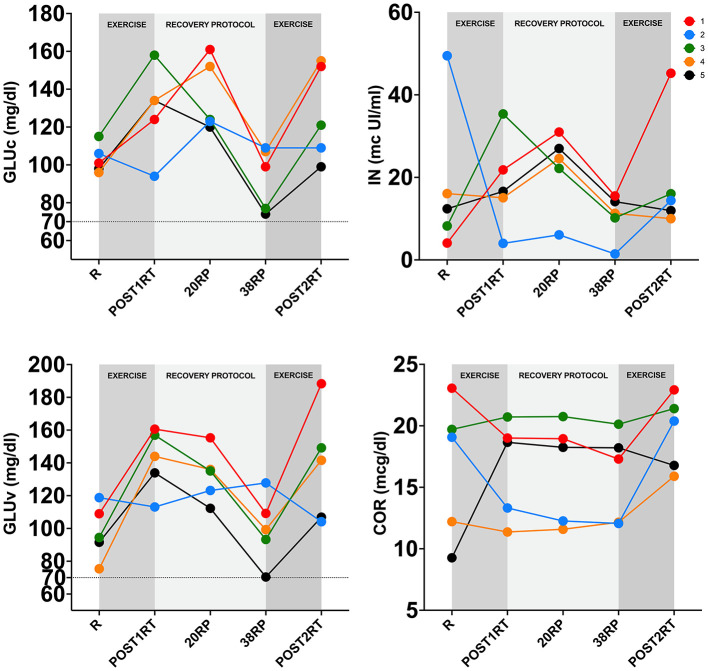
Kinetics of noradrenaline (NA), adrenaline (AD), dopamine (DP), and glucagon (GG) concentrations during the exercise protocol. Data are presented individually.

Capillary (GLUc) and venous (GLUv) glycemic curves showed a parallel temporal pattern throughout the protocol, although with clear differences in peak magnitude and interindividual variability (Table 1 and Figure 2). After the first exercise bout (POST1RT), both compartments reached an initial glycemic peak, with mean values of 129 ± 23 mg/dl for GLUc and 142 ± 19 mg/dl for GLUv. At this time point, venous glycemia was consistently higher than capillary glycemia and was accompanied by a lower coefficient of variation (13.5% vs. 18.0%), indicating a lower observed interindividual dispersion in this sample.

**Table 1:** Changes in capillary and blood glucose, insulin, and cortisol during the exercise protocol, presenting values as a group and individually.

Group values						
		R	POST1RT	20RP	38RP	POST2RT
**GLUc** (mg/dl)	**Mean (SD)**	103 (7.6)	129 (23.1)	136 (19.0)	93 (16.6)	127 (25.3)
	**95% CI**	96–115	94–158	120–161	74–109	99–155
	**CV**	7.4%	18.0%	14.0%	17.8%	19.9%
**GLUv** (mg/dl)	**Mean (SD)**	98 (16.7)	142 (19.2)	132 (16.1)	100 (21.1)	138 (34.6)
	**95% CI**	75–119	113–161	112–155	70–128	104–188
	**CV**	17.1%	13.5%	12.1%	21.1%	25.1%
**IN (**mc UI/ml)	**Mean (SD)**	10.2 (4.5)	18.6 (11.4)	22.2 (9.6)	10.5 (5.5)	19.5 (14.6)
	**95% CI**	4.1–16.1	4.0–35.4	6.1–31.0	1.5–15.5	10.0–45.2
	**CV**	43.9%	61.4%	43.1%	52.3%	74.6%
**COR** (mcg/dl)	**Mean (SD)**	16.7 (5.7)	16.6 (4.0)	16.4 (4.2)	16.0 (3.7)	19.5 (3.0)
	**95% CI**	9.3–23.1	11.4–20.7	11.6–20.8	12.1–20.1	15.9–22.9
	**CV**	34.3%	24.3%	25.4%	23.0%	15.5%
	**Total area**	**Std error**	**IC 95%**			
**AUC GLUc**	473	27.56	419–527			
**AUC GLUv**	492	27.81	437.547			
		R	POST1RT	20RP	38RP	POST2RT
**Individual values**						
GLUc (mg/dl)						
Subject 1		101	124	161	99	152
Subject 2		106	94	123	109	109
Subject 3		115	158	124	77	121
Subject 4		96	134	152	107	155
Subject 5		98	134	120	74	99
GLUv (mg/dl)						
Subject 1		109	161	155	109	188
Subject 2		119	113	123	128	104
Subject 3		95	157	135	93	149
Subject 4		75	144	136	99	142
Subject 5		92	134	112	70	107
IN (mc UI/ml)						
Subject 1		4.1	21.8	31.1	15.5	45.2
Subject 2		49.5	4.0	6.1	1.5	14.4
Subject 3		8.2	35.4	22.2	10.2	16.0
Subject 4		16.1	15.1	24.6	11.3	10.0
Subject 5		12.4	16.6	27.0	14.1	12.0
COR (mcg/dl)						
Subject 1		23.1	19.0	19.0	17.3	22.9
Subject 2		19.1	13.3	12.3	12.0	20.4
Subject 3		19.7	20.7	20.8	20.1	21.4
Subject 4		12.2	11.4	11.6	12.2	15.9
Subject 5		9.3	18.7	18.3	18.2	16.8

Mean (standard deviation). AUC, area under curve; CI, confidence intervals; COR, cortisol; CV, coefficient of variation; GLUc, capillary glucose; GLUv, venous glucose; IN, insulin.

During the recovery phase (Table 1 and Supplementary material), most participants exhibited higher GLUc values at 20RP compared with POST1RT, whereas GLUv generally showed a tendency to decline during the same period. Descriptively, the highest group mean values for GLUc was at 20RP (136 ± 19 mg/dl), while GLUv values were lower than those observed immediately after the first exercise bout (132 ± 16 mg/dl). These observations suggest a potential temporal dissociation between capillary and venous glucose responses during early recovery, although this pattern should be interpreted cautiously given the exploratory nature of the study and the inter-individual variability observed. At 38RP, both glycemic measures declined to values close to or below baseline (GLUc: 93 ± 17 mg/dl; GLUv: 100 ± 21 mg/dl), accompanied by a concomitant increase in variability (CV ≈ 18–21%).

Following the second exercise bout (POST2RT) (Table 1 and Supplementary material), a marked increase in glycemia was observed again in both compartments, reaching values comparable to or higher than those recorded after the first bout (GLUc: 127 ± 25 mg/dl; GLUv: 138 ± 35 mg/dl). At this stage, both compartments coincided in the timing of the peak response; however, interindividual dispersion was high, particularly for venous glycemia (CV = 25.1%), reinforcing the heterogeneous nature of the metabolic response during the final phase of the protocol.

Furthermore, paired AUC values for GLUc and GLUv were compared in an exploratory manner to characterize the magnitude of differences between sampling methods. The observed AUC values were closely aligned (GLUc: 473.0±27.6 vs GLUv: 492.0±27.8; *p* = 0.319) (Table 1 and Supplementary material). Overall, these findings indicate that although capillary and venous glycemic curves follow similar global trends, peak glycemic concentrations do not always occur at the same time points, particularly after the first exercise bout and during the recovery phase.

##### 3.1.1.1 Individual response patterns

The analysis of individual responses revealed generally consistent temporal patterns across participants, although some interindividual variability was observed in the magnitude of the responses (Table 1, Figure 2). For GLUc, four of the five participants showed an increase from rest (R) to POST1RT, followed by maintenance or a slight additional increase at 20RP. Subsequently, all participants demonstrated a reduction at 38RP before increasing again at POST2RT. Despite this common overall pattern, the magnitude of change varied substantially between subjects. Subject 3 exhibited the highest POST1RT value (158 mg/dl), whereas Subject 5 showed the lowest GLUc value at 38RP (74 mg/dl). Importantly, no participant reached hypoglycemic values during the protocol. A similar pattern was observed for GLUv. All participants increased GLUv concentrations from R to POST1RT, with values remaining elevated at 20RP before decreasing at 38RP and rising again at POST2RT. The direction of change was remarkably consistent across participants, although variability in amplitude was evident. Subject 1 demonstrated the highest POST2RT value (188 mg/dl), whereas Subject 5 exhibited the lowest concentration at 38RP (70 mg/dl). Overall, venous glucose responses showed greater consistency between subjects than capillary glucose responses.

#### 3.1.2 Changes in insulin during exercise and recovery

Despite carbohydrate intake during both exercise and recovery, IN exhibited a pattern that was clearly modulated by the exercise stimulus. IN concentrations increased after the first exercise bout (POST1RT: 18.6 ± 11.4 μIU/ml) and reached their highest mean value at 20RT (22.2 ± 9.6 μIU/ml), coinciding with elevated GLUc and GLUv (Table 1 and Figure 2). However, this increase was accompanied by substantial interindividual variability (CV > 43%), reflecting heterogenous insulinemic responses among participants under the same exercise and CHO intake conditions.

During the later phase of recovery (38 RP), IN levels declined to near-baseline values (10.5 ± 5.5 μIU/ml), despite continued carbohydrate intake. This finding indicates a relative exercise-induced suppression of IN secretion and a likely greater contribution of insulin-independent mechanisms to glucose uptake (Table 1 and Supplementary material).

Following the second exercise bout (POST2RT), IN concentrations increased again (19.5 ± 14.6 μIU/ml). Notably, IN exhibited the highest coefficients of variation observed throughout the protocol (CV = 74.6%), indicating that the combination of repeated exercise bouts and carbohydrate intake elicited highly individualized metabolic responses (Table 1 and Supplementary material).

Overall, these results show that IN dynamics varied across the exercise–recovery–exercise sequence under a standardized carbohydrate intake strategy, supporting the feasibility of the protocol to explore GLUv and IN regulation during intermittent exercise.

##### 3.1.2.1 Individual response patterns

Insulin responses showed greater interindividual variability than glucose variables (Table 1, Figure 2). Despite this variability, four participants demonstrated increases in IN concentrations from R to POST1RT and/or 20RP, followed by reductions at 38RP and a subsequent increase at POST2RT. Subject 2 represented the main exception, displaying an atypical pattern characterized by very high resting insulin values (49.5 mcUI/ml) followed by lower concentrations during the remainder of the protocol. In contrast, Subject 1 exhibited the largest increase in insulin, reaching 45.2 mcUI/ml at POST2RT. These findings indicate that, although the temporal trend was generally similar across participants, the magnitude of the endocrine response differed substantially between individuals.

#### 3.1.3 Changes in cortisol during exercise and recovery

COR concentrations remained relatively stable throughout the first exercise bout and the recovery phase, with comparable mean values at R (16.7 ± 5.7 μg/dl), POST1RT (16.6 ± 4.0 μg/dl), 20RT (16.4 ± 4.2 μg/dl), and 38RP (16.0 ± 3.7 μg/dl). At these time points, interindividual variability was moderate, with coefficients of variation ranging from 23.0% to 34.3%, suggesting a relatively homogeneous hypothalamic–pituitary–adrenal axis response during the initial exercise stimulus and the intermediate recovery period (Table 1 and Supplementary material).

Following the second exercise bout (POST2RT), COR levels increased to 19.5 ± 3.0 μg/dl, reaching the highest mean value observed during the protocol. This increase was accompanied by a reduction in the coefficient of variation (15.5%), indicating a more consistent response among participants (Table 1 and Figure 2). The shift of the COR peak toward the final phase of the protocol suggests a response dependent on the duration and/or accumulation of physiological stress, rather than an immediate acute response to the first exercise bout.

##### 3.1.3.1 Individual response patterns

Cortisol concentrations displayed the lowest variability among the measured variables (Table 1, Figure 2). Most participants maintained relatively stable COR values throughout the protocol, with modest increases observed at POST2RT. Subjects 1, 2, and 3 showed slight elevations at the end of the second exercise sector, whereas Subjects 4 and 5 exhibited relatively stable responses across all time points. Overall, cortisol responses suggested a relatively homogeneous endocrine stress response compared with the greater variability observed for insulin.

### 3.2 Catecholamines, dopamine, and glucagon

Catecholamine responses were markedly dependent on the specific phase of the protocol, with clear differences in both peak magnitude and interindividual variability. NA showed a pronounced increase after the first exercise bout (POST1RT: 1,226 ± 700 pg/ml) compared with R (460 ± 175 pg/ml), accompanied by a high coefficient of variation (57.1%), reflecting intense but heterogeneous sympathetic activation. During recovery, NA decreased substantially at 20RP (604 ± 110 pg/ml; CV 18.2%), reaching its lowest variability across the protocol, and then increased again at 38RP (801 ± 360 pg/ml). Following the second exercise bout (POST2RT), NA reached its highest absolute value (2,091 ± 566 pg/ml) with moderate variability (CV 27.1%). This pattern indicates that the greatest sympathetic activation was observed during final phase of the exercise-active recovery-exercise sequence (Table 2 and Supplementary material). AD exhibited a moderate increase after the first exercise bout (POST1RT: 66.0 ± 44.2 pg/ml), followed by a reduction during recovery (20RP: 36.6 ± 16.8 pg/ml; 38RP: 42.4 ± 16.8 pg/ml). However, after the second exercise bout, a marked increase was observed (POST2RT: 115 ± 57 pg/ml), accompanied by high interindividual variability (CV 49.7%), suggesting a greater dependence on the second exercise bout for activation of the sympatho-adrenal axis (Table 2 and Supplementary material).

**Table 2:** Changes in plasma concentrations of norepinephrine, epinephrine, dopamine, and glucagon during the exercise protocol, presenting values as a group and individually.

Group values						
		R	POST1RT	20RP	38RP	POST2RT
**NA** (pg/ml)	**Mean (SD)**	460 (175)	1,226 (700)	604 (110)	801 (360)	2,091 (566)
	**95% CI**	243–698	181–1932	437–740	172–1,048	1,088–2,465
	**CV**	38.0%	57.1%	18.2%	44.9%	27.1%
**AD** (pg/ml)	**Mean (SD)**	47.6 (16.5)	66.0 (44.2)	36.6 (16.8)	42.4 (16.8)	115 (57.2)
	**95% CI**	29.0–73.0	17.0–121.0	20.0–59.0	14.0–57.0	36.0–180.0
	**CV**	34.6%	66.9%	45.9%	39.6%	49.7%
**DP** (pg/ml)	**Mean (SD)**	43.6 (50.1)	86.4 (103.2)	43 (49.5)	39.6 (45.6)	141.6 (126.7)
	**95% CI**	10.0–129.0	12.0–266.0	9.0–130.0	8.0–120.0	24.0–344.0
	**CV**	114.8%	119.5%	115.1%	115.2%	89.5%
**GG** (pg/ml)	**Mean (SD)**	129.4 (3.7)	142.6 (17.3)	123.6 (32.6)	124.8 (33.3)	177.4 (35.2)
	**95% CI**	126.0–135.0	118.0–161.0	103.0–181.0	77.0–168.0	140.0–222.0
	**CV**	2.8%	12.1%	26.4%	26.7%	19.8%
**Individual values**						
NA (pg/ml)						
Subject 1		698	911	740	172	1,088
Subject 2		355	1,391	633	854	2,271
Subject 3		243	1,932	583	1,010	2,344
Subject 4		456	1,717	627	923	2,465
Subject 5		547	181	437	1,048	2,287
AD (pg/ml)						
Subject 1		49	25	20	51	36
Subject 2		38	77	39	42	107
Subject 3		73	121	45	57	180
Subject 4		29	90	59	14	160
Subject 5		49	17	20	48	92
DP (pg/ml)						
Subject 1		48	30	32	17	24
Subject 2		129	266	130	120	344
Subject 3		10	47	16	8	49
Subject 4		15	77	28	28	125
Subject 5		16	12	9	25	166
GG (pg/ml)						
Subject 1		128	152	181	136	190
Subject 2		131	150	104	113	192
Subject 3		135	161	115	168	222
Subject 4		126	132	103	130	140
Subject 5		127	118	115	77	143

Mean (standard deviation). AD, adrenaline; CI, confidence intervals; COR, cortisol; CV, coefficient of variation; DP, dopamine; GG, glucagon; NA, noradrenaline.

DP exhibited high dispersion at all time points (CV > 89%), without a clearly defined pattern comparable to that of NA and AD (Table 2 and Supplementary material). Nevertheless, a trend toward higher values was observed after both exercise bouts, particularly at POST2RT (141.6 ± 126.7 pg/ml), these findings should be interpreted cautiously given the marked interindividual variability and the exploratory nature of the study.

GG increased after the first exercise bout (POST1RT: 142.6 ± 17.3 pg/ml) compared with R (129.4 ± 3.7 pg/ml), decreased during recovery (20RP and 38RP: ~124 pg/ml), and reached its highest value following the second exercise bout (POST2RT: 177.4 ± 35.2 pg/ml) (Table 2 and Supplementary material). GG variability was low at rest (CV 2.8%) and progressively increased with exercise, indicating a consistent counter-regulatory response that was intensified by repeated exercise stimuli.

Overall, these results show that the highest observed values of NA, AD, and GG within the protocol occurred after the second exercise bout, whereas values recorded after the first bout were of lower and accompanied by greater interindividual heterogeneity.

#### 3.2.1 Individual response patterns

**Figure 3:**
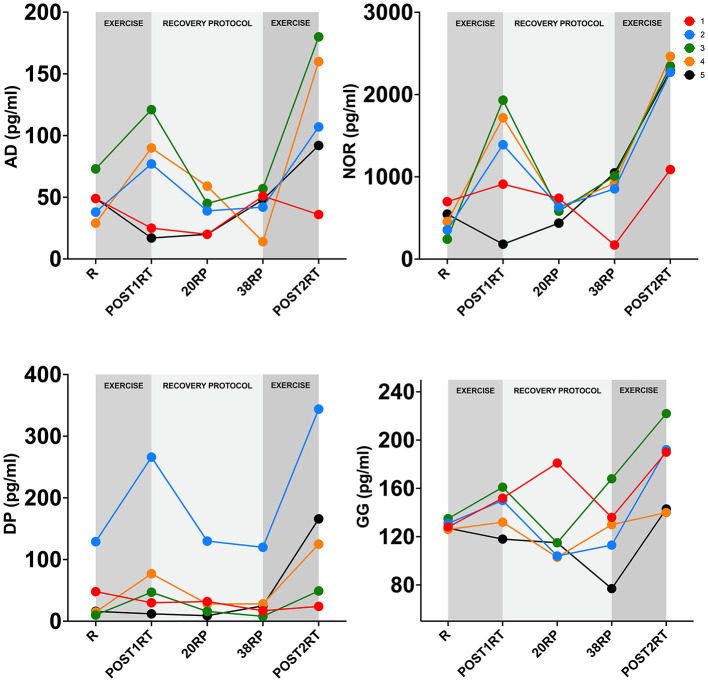
Kinetics of capillary (GLUc) and venous (GLUv) glucose, insulin (IN), and cortisol (COR) concentrations during the exercise protocol. Data are presented individually.

The analysis of individual catecholamine and glucagon responses revealed consistent overall trends across most participants, although marked interindividual variability was observed in the magnitude of several responses, particularly for catecholamines and dopamine (Table 2, Figure 3). For NA, four of the five participants demonstrated a marked increase from rest (R) to POST1RT, followed by a reduction during the recovery phase (20RP and/or 38RP) and a pronounced increase at POST2RT. This general response pattern was particularly evident in Subjects 2, 3, and 4, who exhibited the highest POST2RT concentrations (2,271–2,465 pg/ml). Subject 5 represented the main exception, showing a decrease from R to POST1RT (547 to 181 pg/ml) before increasing progressively during the remainder of the protocol and reaching 2,287 pg/ml at POST2RT. Despite differences in magnitude, all participants showed their highest or near-highest NA values at the end of the second exercise sector.

AD responses were more heterogeneous than NA responses (Table 2, Figure 3). Three participants (Subjects 2, 3, and 4) exhibited increases from R to POST1RT, whereas Subjects 1 and 5 showed decreases during the same period. During the recovery phase, AD concentrations generally decreased or remained stable before increasing again at POST2RT in four participants. Subject 3 displayed the highest overall AD response, reaching 180 pg/ml at POST2RT. In contrast, Subject 1 maintained relatively low AD concentrations throughout the protocol, with minimal changes between time points.

DP showed the greatest interindividual variability of all hormonal variables (Table 2, Figure 3), as reflected by the consistently elevated coefficients of variation (>89%). Subjects 2 and 4 demonstrated progressive increases from R to POST2RT, reaching peak concentrations of 344 pg/ml and 125 pg/ml, respectively. Subject 5 also exhibited a marked increase at POST2RT despite low concentrations throughout the previous phases. Conversely, Subjects 1 and 3 maintained relatively low DP concentrations during the entire protocol, with only modest increases at POST2RT. Although the magnitude of response differed substantially, most participants demonstrated higher DP values at POST2RT compared with resting conditions.

GG responses showed a more homogeneous temporal pattern across participants than catecholamines (Table 2, Figure 3). Four participants demonstrated increases from R to POST1RT, followed by either stabilization or slight reductions during the recovery phase, and a subsequent increase at POST2RT. Subjects 1, 2, and 3 showed the highest GG concentrations at POST2RT (190–222 pg/ml). Subject 5 was the main exception, displaying a progressive reduction from POST1RT to 38RP before a modest increase at POST2RT. Overall, most participants exhibited higher glucagon concentrations during the second exercise sector compared with resting conditions and the first exercise sector.

### 3.3 Lactate, lipids, body mass, and perceived exertion

LAC concentration exhibited a clearly biphasic pattern closely associated with the exercise bouts. After the first bout (POST1RT), LAC increased from 2.1 ± 0.6 mmol/L to 4.1 ± 1.3 mmol/L (CV 33.0%). During recovery, LAC decreased at 20RT (2.7 ± 1.7 mmol/L), albeit with high variability (CV 63.0%), and increased again at 38RT (3.5 ± 1.2 mmol/L). Following the second exercise bout, LAC reached the highest value of the protocol (POST2RT: 4.2 ± 1.0 mmol/L; CV 22.7%), illustrating the temporal dynamics of LAC across the protocol (Table 3 and Supplementary material).

**Table 3:** Changes in capillary blood lactate concentrations and plasma free fatty acids and triglycerides, perceived exertion, and body weight during the exercise protocol, presenting values as a group and individually.

Group values						
		R	POST1RT	20RP	38RP	POST2RT
**BM** (Kg)	**Mean (SD)**	71.9 (10.1)	71.3 (10.0)	71.3 (9.9)	71.3 (9.9)	70.9 (10.0)
	**95% CI**	60.9–85.7	60.4–84.9	60.4–84.7	60.4–84.7	59.8–84.1
	**CV**	14.0%	14.0%	13.9%	14.0%	14.1%
**LAC** (mmol/L)	**Mean (SD)**	2.1 (0.6)	4.1 (1.3)	2.7 (1.7)	3.5 (1.2)	4.2 (1.0)
	**95% CI**	1.4–2.9	2.3–5.6	1.7–5.8	2.2–5.3	2.9–5.9
	**CV**	27.9%	33.0%	63.0%	33.3%	22.7%
**RPE**	**Mean (SD)**	0.0 (0.0)	7.5 (0.6)	2.1 (0.7)	6.7 (1.0)	8.1 (1.0)
	**95% CI**	0.0–0.0	7.0–8.5	1.0–3.0	5.5–8.5	6.5–9.0
	**CV**	8.2%	35.3%	14.6%	11.9%	8.2%
**TRI** (mg/dl)	**Mean (SD)**	93 (18.5)	110 (40.1)	90 (29.2)	101 (41.5)	109 (40.3)
	**95% CI**	60.7–106.8	58.7–170.4	49.4–131.0	53.0–166.5	64.2–173.9
	**CV**	20.0%	36.5%	32.3%	41.2%	36.9%
**FFA** (mmol/L)	**Mean (SD)**	0.98 (0.17)	0.58 (0.01)	0.24 (0.24)	0.20 (0.21)	0.73 (0.58)
	**95% CI**	0.03–0.30	0.13–1.27	0.04–0.59	0.05–0.53	0.14–1.61
	**CV**	118.0%	79.2%	101.4%	104.4%	79.3%
BM (Kg)						
Subject 1		85.7	84.9	84.7	84.7	84.1
Subject 2		60.9	60.4	60.4	60.4	59.8
Subject 3		76.2	75.8	75.9	75.9	75.9
Subject 4		63.4	62.8	63.0	62.7	62.4
Subject 5		73.3	72.7	72.7	72.7	72.3
LAC (mmol/L)						
Subject 1		1.8	5.2	1.7	3.3	2.9
Subject 2		1.9	2.3	2.3	3.7	4.4
Subject 3		1.4	3.9	2.1	2.9	4.5
Subject 4		2.4	3.4	1.8	2.2	3.8
Subject 5		2.9	5.6	5.8	5.3	5.5
RPE
Subject 1		0.0	7.5	2.0	7.0	6.5
Subject 2		0.0	7.0	3.0	8.0	8.0
Subject 3		0.0	7.0	1.0	6.0	8.5
Subject 4		0.0	7.5	2.5	5.5	8.5
Subject 5		0.0	8.5	2.0	7.0	9.0
TRI (mg/dl)						
Subject 1		98.1	109.2	92.5	87.7	102.5
Subject 2		103.7	170.4	131.0	166.5	173.9
Subject 3		94.2	98.4	83.3	91.2	111.1
Subject 4		106.8	113.0	95.7	105.5	93.9
Subject 5		60.7	58.7	49.4	53.0	64.2
FFA (mmol/L)						
Subject 1		0.300	1.270	0.590	0.530	1.610
Subject 2		0.040	0.130	0.080	0.070	0.140
Subject 3		0.090	0.830	0.390	0.300	0.910
Subject 4		0.030	0.390	0.040	0.060	0.290
Subject 5		0.030	0.300	0.090	0.050	0.710

Mean (standard deviation). BM, body mass; CI, confidence intervals; CV, coefficient of variation; FFA, free fatty acids; LAC, lactate; RPE, rate of perceived exertion; TRI, triglycerides.

Body mass decreased gradually throughout the protocol, from 71.9 ± 10.1 kg at R to 70.9 ± 10.0 kg at POST2RT, with stable coefficients of variation (~14%). This progressive reduction is compatible with acute changes in whole-body mass commonly observed during prolonged exercise (Table 3 and Supplementary material), under conditions of sustained metabolic and thermoregulatory demand. Although fluid loss likely contributed to this pattern, body mass was used here as an indirect indicator rather than a direct measure of hydration status. Given the controlled fluid intake, losses did not exceed 2% of initial body mass, a threshold often considered physiologically relevant. Other contributors, such as measurement variability and minor mass loss related to substrate oxidation and respiratory water loss, cannot be excluded and may have contributed to the observed changes.

Free fatty acids (FFA) showed a marked reduction after the first exercise bout (POST1RT: 0.58 ± 0.01 mmol/L) compared with R (0.98 ± 0.17 mmol/L), reaching their lowest values during recovery (20RP: 0.24 ± 0.24 mmol/L; 38RP: 0.20 ± 0.21 mmol/L). After the second exercise bout, FFA increased again (POST2RT: 0.73 ± 0.58 mmol/L), although with very high variability (CV 79.3%), suggesting highly individualized lipolytic responses (Table 3 and Supplementary material).

Plasma triglycerides showed modest changes without a clear temporal pattern, with increases after both exercise bouts (POST1RT: 110 ± 40 mg/dl; POST2RT: 109 ± 40 mg/dl) and decreases during recovery, in all cases accompanied by considerable variability (CV 32–41%) (Table 3 and Supplementary material).

Ratings of perceived exertion (RPE) closely mirrored the pattern of LAC and intermittent exercise, with high values after both bouts (POST1RT: 7.5 ± 0.6; POST2RT: 8.1 ± 1.0) and a marked reduction during recovery (20RP: 2.1 ± 0.7). Notably, coefficients of variation were low at the points of highest effort (< 12%), indicating a consistent perceptual response among participants (Table 3 and Supplementary material).

#### 3.3.1 Individual response patterns

**Figure 4:**
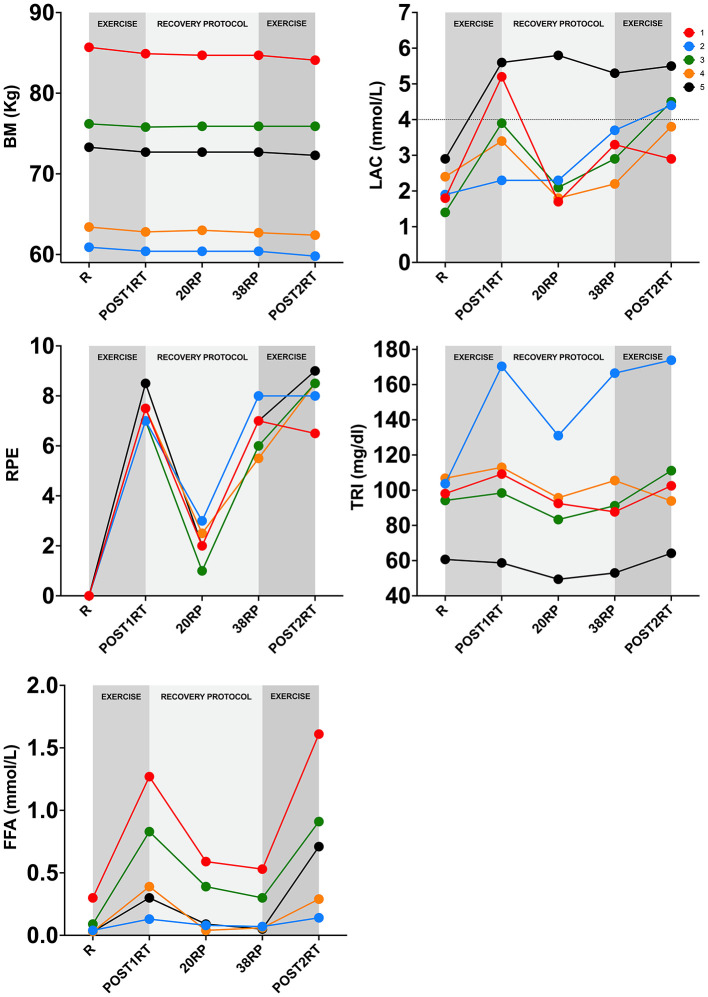
Kinetics of body weight (BM), perceived exertion (RPE), and concentrations of lactate (LAC), triglycerides (TRI), and free fatty acids (FFA) during the exercise protocol. Data are presented individually.

The individual analysis of body mass, metabolic markers, and perceptual responses demonstrated relatively consistent temporal patterns across participants, although variability in the magnitude of the responses was observed for some variables, particularly FFA and triglycerides (Table 3, Figure 4). For BM, all participants exhibited either stable values or progressive reductions throughout the protocol. Four participants (Subjects 1, 2, 4, and 5) showed gradual decreases from rest (R) to POST2RT, whereas Subject 3 maintained virtually unchanged body mass throughout the protocol. Importantly, no participant exhibited body mass reductions exceeding 2% of initial values, indicating that hydration status was generally preserved during the exercise protocol (Table 3, Figure 4).

LAC responses showed a relatively consistent pattern characterized by increases after the exercise sectors and partial reductions during the recovery phase (Table 3, Figure 4). All participants increased LAC concentrations from R to POST1RT. Subsequently, four participants demonstrated reductions at 20RP before increasing again at 38RP and/or POST2RT. Subject 5 represented the main exception, maintaining consistently elevated LAC concentrations throughout the entire protocol, with values ranging from 5.3 to 5.8 mmol/L during the recovery and second exercise phases. Subjects 2, 3, and 4 exhibited progressive increases from 38RP to POST2RT, whereas Subject 1 showed a slight reduction at POST2RT compared with 38RP.

RPE responses were highly consistent across participants and closely paralleled exercise intensity (Table 3, Figure 4). All participants reported substantial increases in RPE at POST1RT, marked reductions during the recovery phase (20RP), and renewed increases during the later stages of the protocol, with the highest values generally observed at POST2RT. Subject 5 reported the highest perceived exertion values throughout the protocol, reaching an RPE of 9.0 at POST2RT, whereas Subject 1 displayed the lowest POST2RT RPE value (6.5). Despite minor differences in magnitude, all participants demonstrated similar perceptual response patterns across the exercise protocol.

TRI concentrations showed moderate interindividual variability (Table 3, Figure 4). Subjects 1, 2, 3, and 4 demonstrated increases from R to POST1RT, whereas Subject 5 showed a slight reduction during the same period. During the recovery phase, most participants exhibited either stabilization or modest reductions in TRI concentrations before increasing again at POST2RT. Subject 2 consistently displayed the highest TRI values throughout the protocol, reaching 173.9 mg/dl at POST2RT, while Subject 5 maintained the lowest concentrations across all time points.

FFA responses demonstrated the greatest interindividual variability among the metabolic variables (Table 3, Figure 4). Despite this variability, all participants showed an increase from R to POST1RT, followed by reductions during the recovery phase (20RP and 38RP). Subsequently, all participants increased FFA concentrations again at POST2RT. Subject 1 exhibited the most pronounced response, with FFA values increasing to 1.610 mmol/L at POST2RT, whereas Subjects 2 and 4 maintained comparatively low concentrations throughout the protocol. Although the magnitude of the response differed markedly between individuals, the temporal pattern of an initial increase, recovery-associated reduction, and final increase was generally preserved across participants.

### 3.4 Heart rate

Heart rate (HR) exhibited a clearly differentiated response between the exercise bouts and the recovery phases (Table 4 and Supplementary material). From baseline values of 82.2 ± 15.6 bpm, HR increased acutely after the first exercise bout (POST1RT: 172.6 ± 13.6 bpm), with low interindividual variability (CV 7.9%). During recovery, HR declined rapidly at 10RT (118.4 ± 23.4 bpm) and 20RT (114.0 ± 5.2 bpm), reaching the lowest coefficient of variation of the protocol at 20RT (CV 4.5%), indicating a highly consistent cardiovascular recovery response among participants. As recovery progressed, HR showed a slight rebound at 30RT (122.4 ± 37.5 bpm) and a more pronounced increase at 38RT (156.0 ± 23.5 bpm), anticipating the onset of the second exercise bout. After the second bout (POST2RT), HR reached the highest value of the protocol (178.0 ± 13.0 bpm), again with low variability (CV 7.3%). The temporal coincidence of maximal HR peaks after both exercise bouts, together with the rapid and homogeneous reduction during recovery, confirms adequate physiological separation between protocol phases and supports the feasibility of the design for investigating cardiovascular responses to high-demand intermittent exercise.

**Table 4:** Changes in heart rate during the exercise protocol, presenting values as a group and individually.

		R	POST1RT	10RP	20RP	30RP	38RP	POST2RT
**HR** (bpm)	**Mean (SD)**	82.2 (15.6)	172.6 (13.6)	118.4 (23.4)	114.0 (5.2)	122.4 (37.5)	156.0 (23.5)	178.0 (13.0)
	**95% CI**	65.0–100.0	160.0–195.0	87.0–153.0	109.0–122.0	70.0–158.0	120.0–185.0	159.0–195.0
	**CV**	19.0%	7.9%	19.8%	4.5%	30.6%	15.1%	7.3%
HR (bpm)								
Subject 1		71	160	87	109	129	120	159
Subject 2		65	164	117	111	70	152	177
Subject 3		78	195	153	122	158	185	195
Subject 4		97	171	120	116	155	161	183
Subject 5		100	173	115	112	100	162	176

Mean (standard deviation). CI, confidence intervals; CV, coefficient of variation; HR, heart rate.

#### 3.4.1 Individual response patterns

**Figure 5:**
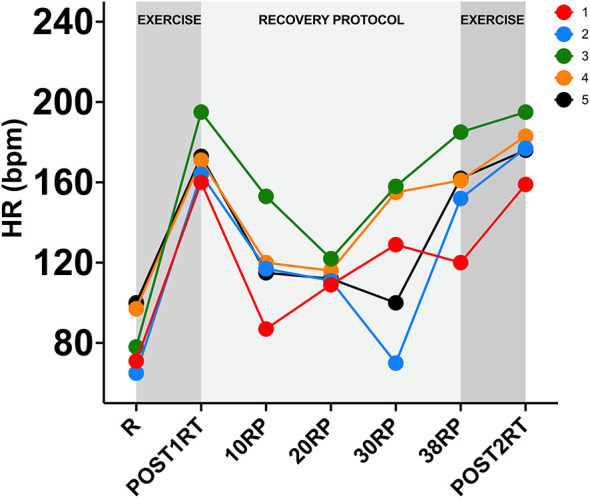
Protocol used to evaluate changes in hormonal, metabolic and dehydration markers. CH = carbohydrates.

Analysis of the individual HR responses revealed a highly consistent cardiovascular pattern across the five participants throughout the protocol (Table 4, Figure 5). All subjects showed a marked increase in HR from rest (R) to the end of the first rectangular test (POST1RT), reaching values compatible with the target intensity of ~90% HR_MAX_. Subsequently, during the recovery phase (RP), all participants demonstrated a reduction in HR at 10RP and 20RP, confirming the partial recovery effect induced by the lower-intensity segment performed at 60% HR_MAX_. Despite this general decrease during RP, inter-individual variability became more evident at 30RP and 38RP, coinciding with the progressive increase in exercise intensity and the inclusion of shuttle runs and short high-intensity bouts. Subjects 3 and 4 exhibited the highest HR values during these stages (158–185 bpm and 155–161 bpm, respectively), whereas Subject 2 showed a transient reduction at 30RP (70 bpm), representing the main individual deviation from the overall group trend. Nevertheless, HR increased again in Subject 2 at 38RP and POST2RT, indicating recovery of the expected cardiovascular response.

At the end of the second rectangular test (POST2RT), all participants again reached high HR values (159–195 bpm), demonstrating a reproducible cardiovascular strain during both exercise sectors. Subjects 3 and 4 consistently displayed the highest HR responses across the protocol, whereas Subjects 1 and 2 tended to present lower absolute HR values despite maintaining the same temporal response pattern. Overall, the individual analysis supports a homogeneous internal load across participants, with all subjects exhibiting the expected increase–recovery–increase HR sequence throughout the protocol.

## 4 Discussion

The main objective of this study was to evaluate how the different hormones involved in GLUv regulation interact during an exercise protocol that was designed to mimic, from a physiological and nutritional standpoint, the one to be performed by racewalkers in the mixed relay event at the Olympic Games Paris 2024, where they ingested 50 g of CH in 500 ml of water in each phase of the protocol (1st exercise, recovery and 2nd exercise) and a gel of 45 g of CH in the recovery phase. Given the pilot and feasibility nature of the study (*n* = 5), the discussion is focused on describing physiological trends and response patterns rather than establishing definitive cause–effect relationships.

The present results should therefore be interpreted primarily from a descriptive and exploratory perspective, with particular emphasis on the consistency or heterogeneity of individual responses across participants rather than on definitive group-level conclusions.

Accordingly, the most relevant observation is not the magnitude of group mean responses, but the consistency of intra-individual temporal patterns, particularly the repeated sequence of increase–recovery–increase across GLUv, GLUc, HR, and several hormonal markers. Across participants, the dominant response pattern was characterized by a reproducible elevation of GLUv, GLUc, insulin, and catecholaminergic markers during exercise phases, followed by partial normalization during recovery and a subsequent re-elevation during the second exercise bout. However, the amplitude of these responses was markedly individual-specific, especially for insulin and free fatty acids, indicating heterogeneous endocrine sensitivity to identical external load and nutritional conditions.

Importantly, despite the ingestion of ~95 g CH during recovery, no participant developed hypoglycemia prior to the second exercise bout, and both GLUv and GLUc remained within normoglycemic ranges across all subjects. However, post-exercise elevations in plasma FFA were still observed, indicating that carbohydrate intake did not fully suppress lipolytic activity in all individuals, consistent with preserved sympathetic drive and variable insulin responsiveness.

Furthermore, although the exercise modality in our protocol was running, the relative physiological load imposed was designed to approximate the internal demands experienced by competitive racewalkers. Research comparing competitive walking and running has demonstrated that physiological responses, including heart rate, ventilation, perceived exertion, and LAC concentration, can be remarkably similar between the two modalities when performing submaximal and maximal effort when exercise intensity matches oxygen consumption (VO_2_) (56). This supports the use of relative intensity (e.g., % HR_MAX_ or % VO_2_MAX__) as a common framework for comparing internal physiological stress between race walking and running, even though the biomechanical pattern is different.

### 4.1 Hormonal and metabolic changes in the first phase of the exercise protocol

During the first exercise sector (B-POST1RT; 40 min at 90% HR_MAX_) and with an intake of 50 g CH, an elevation of GLUv, GLUc and IN was observed in POST1RT, although the GLUv peak was earlier than that of GLUc. The predominant pattern was an increase in GLUv, GLUc, and insulin at POST1RT, although individual trajectories diverged considerably in insulin response amplitude. In agreement with our data, other authors have reported an increases of GLUv concentration following endurance exercise of comparable duration and intensity (60%-90% VO_2_MAX__ for 40-60 min) with CH intake (solution: 6–10% glucose or maltodextrin) (57–60). This increase in GLUv despite high muscular glucose utilization reflects the capacity of hepatic glucose production to match or exceed peripheral glucose uptake during high-intensity exercise. This process is primarily mediated by GG and catecholamines stimulation of hepatic glycogenolysis, with additional contributions from gluconeogenesis using lactate, glycerol and amino acids as substrates (7, 61, 62). The elevated GLUv observed in the presented study likely reflects the combined contribution of exogenous glucose absorption and increased hepatic glucose output, ensuring adequate substrate availability for working skeletal muscle and the central nervous system.

However, Below et al. (58) found an elevation of GLUv in both the CH (6% maltodextrin solution: 79g CH) and PLA groups during a test at 80% VO_2_MAX__ (50 min) plus a performance test (amount of work in the shortest possible time). This observation suggests that exercise-induced hepatic glucose output can independently maintain glycemia even in the absence of CHO ingestion, although exogenous CHO intake enhances glucose availability and reduces reliance on endogenous glycogen stores (63, 64). This mechanism is particularly relevant in repeated exercise scenarios, where glycogen preservation during the first exercise bout may improve metabolic stability in subsequent bouts. At the start of high-intensity exercise without prior intake or during exertion, hepatic glucose production increases mainly due to increased glycogenolysis (65–68), together with an additional contribution from neoglucogenesis, in which La^−^ acts as one of the main substrates (≈20% of the rate of appearance in blood during the first 35–50 min at 40% of VO_2_PEAK__) (69). However, glycogenolysis predominates over gluconeogenesis, being greater at higher intensities, with 62% higher La^−^ uptake but with a lower relative contribution to neoglucogenesis (68).

In addition, we no found changes in NA and GG in POST1RT. Mitchell et al. (70) evaluated how CH intake (33.5, 39.5, and 50.1 g/h) modulated GLUv, IN, GG, AD, and NA levels during a 70% VO_2*MAX*_ test on a cycle ergometer for 115 min. This author found higher GLUv concentrations with different amounts of CH at 25 and 55 min compared to rest; an increase in IN only in the group that ingested 50.1 g/h of CH at 25 min compared to rest; no changes in GG at 25 and 55 min, although higher GG levels were observed in the PLA group at 85 and 115 min compared to the group that consumed 50.1 g/h of CH (70). The absence of de substantial increase in counterregulatory hormones during the first exercise sector likely reflects adequate CHO availability and preserved glycemic homeostasis. Counterregulatory hormonal responses are primarily activated when glucose availability becomes insufficient relative to metabolic demand, rather than being triggered solely by exercise intensity (30, 71). Therefore, the CHO intake strategy used in this study may have attenuated the need for strong endocrine counter regulation during the initial exercise bout.

Specifically, the increases in NA and A in the first sector of our study. This may be because A responds mainly to situations of severe hypoglycemia (72), and NA is not a potent counter-regulatory hormone (73, 74). Therefore, from a feasibility perspective, the selected strategy of fluid and carbohydrate intake (50 g CH/500 ml/40 min) in the first sector of the protocol appears to promote metabolic stability.

Furthermore, from rest (R) to POST1RT, they observed parallel increases in LAC concentrations, FFA, HR, RPE, and a decrease in BM (−0.8%). These temporal patterns were generally reproduced across the five participants, although FFA responses displayed marked interindividual variability. Contrary to our results, the findings reported by El-Sayed et al. (75), where after administering 25 g of CH pre- and post-exercise (87% VO_2_MAX__ in 1 h), FFA levels were similar during and at the end of the test, but there was an increase in FFA in both the PLA and CH groups 15 and 30 min after the end of the test. Furthermore, in another subsequent study in which subjects ingested a higher amount of CH (31 g), it was shown that CH intake at the end of submaximal exercise (70% of VO_2_MAX__ in 90 min) promoted a higher concentration of FFA at that time and in the subsequent recovery period (76). The increase in FFA despite CHO intake likely reflects sustained sympathetic activation and incomplete suppression of lipolysis. Catecholamines stimulates lipolysis through activation of hormone-sensitive lipase, allowing simultaneous CHO and lipid utilization during high-intensity endurance exercise (2, 77). This metabolic flexibility is characteristic of endurance-trained individuals and contributes to maintaining energy supply during prolonged exercise.

In addition, the fluid intake protocol prevented dehydration, as only −0.8% of BM was lost in the first 40 min of the exercise protocol, with cautionary data due to the small sample size. In this regard, avoiding dehydration of more than 2% of body weight worsens aerobic and cognitive performance, and at higher levels of dehydration, the negative effect on performance and physical fitness is greater (78). This combination of cardiovascular and perceptual strain, together with preserved metabolic homeostasis, supports the adequacy of the protocol to induce high physiological stress without compromising glycemic control in this initial phase.

### 4.2 Hormonal and metabolic changes in the second phase (recovery phase) of the exercise protocol

In the RP (20 min at 60% of HR_MAX_ (20RP) + 8 min at 85% of HR_MAX_ + 3 sets of 10 m running back and forth with elastic bands + 2 sets of 2 min at 90% of HR_MAX_ (38RP)), we observed a decrease in GLUv and IN from POST1RT to 38RP. In addition, we found a decrease in HR from POST1RT to 20RP and an increase in HR from 20RT to 38RT, with similar evolution in LAC. Most participants demonstrated this recovery-associated reduction followed by a subsequent increase toward the second exercise sector, supporting the reproducibility of the protocol-induced physiological responses. Our results coincide with those found by El-Sayed et al. (75, 76), where after performing a 90 min test at 70%VO_2_MAX__ and 1h trial time (TT) at 87%VO_2_MAX__, at 10 and 15 min there was a decrease in LAC and HR in the recovery phase, but the methodologies used were different both in the intensity of the exercise protocol and the amount of CH ingested. Specifically, El-Sayed et al. (76), used an intake of 28 g 15 min before, 20 g before starting and 20 g of CH at the end of a test at 70%VO_2_MAX__, where they observed a decrease in GLUv (99 to 59 mg/dl) from the beginning to the end of exercise, contrary to our results (98 to 142 mg/dl), since in our study 50 g of CH were ingested. On the other hand, in the study by El-Sayed et al. (76) after the end of the exercise protocol, there is an elevation of GLUv (54 to 70 mg/dl) at 10 min in the recovery phase (at rest) and stabilization until 30 min. This decrease in both HR and GLUv likely reflects the combined effects of lower exercise intensity, increased glucose uptake by skeletal muscles for glycogen resynthesis, and insulin-mediated glucose disposal following carbohydrate intake (38, 79). After exercise, skeletal muscle exhibits enhanced insulin sensitivity, which accelerates glucose uptake and glycogen resynthesis, even during active recovery (80, 81). Importantly, this heterogeneity was more pronounced than during exercise phases, suggesting that recovery represents a phase of higher endocrine inter-individual variability than active exercise under standardized conditions.

One year later, El-Sayed et al. (75) observed how an intake of 25 g CH or PL before and after 1h of TT did not prevent a decrease (75 to 47 mg/dl) of GLUv after the end of the exercise protocol (87%VO_2_MAX__) in both groups. In addition, this author also found an exponential GLUv increase at 15 min and 30 min after recovery (at rest). In addition, another author evaluated the effect of high (349 g/4h) or low (87 g/4h) CH intake in recovery (4h at rest) from exercise at 70%VO_2_MAX__ to exhaustion on the performance of a subsequent exercise at the same intensity (82). In this study despite drinking only water during the exercise protocol a stable GLUv was maintained, but in recovery the group that ingested a high CH intake had a rapid increase in GLUv in the 1 h (93 to 126 mg/dl) and steep decline (126 to 90 mg/dl) until the beginning of the second exercise, however, the group that ingested a low CH intake had a progressive increase (93 to 115 mg/dl) in the recovery phase. In addition, the group that had high CH intake (1.2 g CH·kg^−1^·h^−1^) in recovery had higher muscle glycogen resynthesis and higher performance (until exhaustion) in a second exercise compared to low CH intake (0.3 g CH·kg^−1^·h^−1^) (82). Muscle glycogen resynthesis rates are maximized when CHO intake exceeds approximately 1.0–1.2 g·kg^−1^·h^−1^ during early recovery, primarily due enhanced insulin action and increased GLUT-4 activity (38, 83). Although muscle glycogen was not directly measured in the present study, the maintenance of glycemia and insulin levels suggests favorable conditions for glycogen restoration.

Nevertheless, some variables such as insulin and catecholamines showed substantial interindividual dispersion during recovery, suggesting that endocrine regulation during this phase may be particularly individualized even under standardized nutritional conditions.

Therefore, consuming a high intake of HC can promote greater increases in GLUv with increases in IN compared to low CH intakes, leading to a more optimal way of maintaining GLUv levels and higher rates of muscle glycogen generation at the end of the recovery period.

Moreover, BM was maintained during RP, this phase of the protocol evaluated in this study is based on an active recovery with gradual intensity increase until the second exercise sector (40 min work at 90% of HR_MAX_). This preservation of hydration likely contributed to maintaining cardiovascular stability and optimal glucose delivery, as dehydration can impaired muscle perfusion and glucose transport (84, 85). Therefore, the intake of 500 ml of water with 50 g of CH was sufficient to maintain BM.

### 4.3 Hormonal and metabolic changes in the third phase of the exercise protocol

In the second exercise sector (38RP-POST2RT; 40 min at 90% HR_MAX_) of the evaluated protocol, we observed elevation of the HR, GLUv, IN, and FFA. At the individual level, this phase elicited the greatest divergence in endocrine and metabolic responses, particularly for catecholamines and glucagon. However, Alghannam et al. (82), where after performing an exercise at 70%VO_2_MAX__ until exhaustion, followed by a 4h recovery with high CH intake (349 g/4h) and then the study subjects performed another exercise at 70%VO_2_MAX__ until exhaustion. They detected an increase in GLUv (74 to 94 mg/dl) and non-esterifiable fatty acids (reaching values of 0.732 mmol/L) with a maintenance of IN levels (82). The differences between this study and ours were the intensity of exercise and the intake of CH during exercise, specifically in this study no CH was ingested, whereas in ours 50 g CH was ingested in 500 ml of water. These differences in CH intake and exercise intensity could lead to observe different IN patterns in the second exercise sector of their respective evaluated protocol. These responses reflect the progressive activation of neuroendocrine mechanism to maintain glucose availability during repeated exercise. Repeated exercise bouts can lead to cumulative sympathetic activation, resulting in increased catecholamine release, which stimulates hepatic glucose production and adipose tissue lipolysis (30, 31). Importantly, the magnitude of this activation was strongly participant-dependent, suggesting inter-individual variability in sympathetic and glucoregulatory sensitivity under repeated high-intensity stress.

At the end of the two exercises of our evaluated protocol the values of GLUv (POST1RT: 142 vs POST2RT: 138 mg/dl) and IN (POST1RT: 18.6 vs POST2RT: 19.5 mcUI/ml) were similar. In addition, we observed an increase in GG, NA, AD, and COR. However, this apparent group stability masked individual variability, as some participants exhibited higher second-bout counter-regulatory activation despite similar glycemic levels. This indicates higher concentrations of counterregulatory hormones in the second exercise period compared to the first. The increase in GG may be due to a decrease in GLUv (86), not our case, but it has also been postulated that catecholamines (increase in NA in our study) promote a strong stimulatory effect on GG secretion. (87–89). On the other hand, the increase in GG could be due to an increase in hepatic glucose production by stimulating both hepatic gluconeogenesis and glycogenolysis, increasing both the extraction of neoglycogen precursors by the liver and the conversion of precursors to glucose at the hepatic level (90). This stability suggest that exogenous CHO intake successfully compensated for increased glucose utilization, preventing progressive hypoglycemia. Nevertheless, individual responses indicate that this compensation was achieved through different hormonal strategies across participants. This finding supports previous research demonstrating that CHO intake during prolonged exercise helps maintain glycemic stability and delays fatigue (63, 64). In addition, the increase in counterregulatory hormones during the second exercise sector likely reflects cumulative physiological stress and progressive sympathetic activation. This increase was not homogeneous across participants, with glucagon and catecholamines showing particularly high inter-individual variability. Catecholamines stimulate hepatic glucose production and reduce insulin-meditated glucose uptake in non-exercising tissues, prioritizing glucose delivery to active skeletal muscle and the central nervous system (7, 71). Additionally, cortisol contributes to maintaining glucose availability by stimulating gluconeogenesis and enhancing lipolysis, particularly during prolonged or repeated exercise (25). The moderate increase observed in this study likely reflects the combined influence of exercise intensity and cumulative metabolic stress.

Furthermore, there was a loss of BM (−0.6%) in the second sector of the exercise, with a decrease of 1.4% in BM throughout the entire protocol evaluated. As already known from previous studies, BM losses above this threshold (>2% BM) in prolonged exercise produce high sweating rates leading to meaningful body fluid losses (91–93). This situation can decrease cardiac output, alter cutaneous and central thermoregulatory function, increase perceived exertion, decrease blood flow to the muscle, and negatively affect endurance exercise performance (91). To prevent deterioration of cardiovascular function and performance, electrolyte and CH drinks are widely recommended to reduce the risk of dehydration (93) and hyponatremia (92). Maintaining hydration likely prevented excessive cardiovascular strain and endocrine disruption, supporting metabolic stability during repeated exercise (85).

It is known that environmental factors, hydration levels, and water balance affect metabolic and endocrine responses to prolonged exercise. In thermoneutral environments, dehydration of more than 2% of body mass has been linked to an increase in cardiovascular effort, an elevated central temperature, and changes in substrate metabolism (e.g., elevated catecholamines levels and altered glucose metabolism) (94, 95). In the current study, the amount of liquids consumed was standardized at 500 ml with carbohydrates administered in each phase, which led to a decrease in body mass of less than 2% throughout the entire procedure. This level of fluid loss is below the thresholds typically associated with an important change in cardiovascular and metabolic function, suggesting that the maintenance of hydration may have contributed to the relatively stable GLUv and IN responses observed.

Briefly, with respect to potential performance—related implications, the present finding descriptively indicate that the intake of 50g CH/40 min/500 ml in each phase of the protocol plus a gel (45 g CH) in the recovery phase with a total of 195 g CH with 1,500 ml ingested, was associated with the maintenance of glycemic levels and body mass within a stable range throughout the protocol. Given that hypoglycemia and dehydration are commonly recognized physiological stressors during prolonged endurance exercise, these observations may be relevant for the design of future performance—focuses studies. At the hormonal level, exercise at 90% of HRmax did not produce changes in IN during both exercise sectors despite CH intake. During recovery the high intake of CH did not produce hypoglycemia, since the minimum value of GLUv was 93 mg/dl. The magnitude of increase in GLUv was similar in both exercise sectors, however, the responses of counter-regulatory hormones (NA and GG) were higher in the second exercise sector than the first.

### 4.4 Limitations

The main limitation of this study lies in the small sample size (*n* = 5), which limits statistical power and prevents generalizable inferential conclusions from being drawn. Therefore, this study should be interpreted strictly as a descriptive pilot study, designed to generate hypotheses and identify physiological trends, rather than to confirm effects using inferential statistics. In descriptive studies with small samples, *p*-values tend to underestimate actual physiological effects due to inter-individual variability and insufficient power, which can lead to type II errors. The sample does not correspond to racewalkers, clarifying that, although the relative metabolic intensity is comparable between modalities when the internal load is controlled, direct extrapolation to competitive performance in race walking should be done with caution, but our study does not aim to evaluate performance. Future research with larger samples size to increase statistical power and allow inferential analyses, compare alternative CH doses, composition, and timing strategies. Moreover, examine alternative hydration strategies and environmental conditions, include direct performance outcome and muscle glycogen measurements and explore inter-individual variability in hormonal a metabolic response. Is needed to validate and extend these findings, especially given the growing relevance of the mixed-race walking relay format, which is expected to reappear at the 2028 Olympic Games in Los Angeles.

### 4.5 Practical applications

This pilot study provides useful information for hydration and CH intake strategies in high-intensity intermittent endurance sports, such as mixed relay racewalking, which combines prolonged efforts with short breaks. Intake of 50 g of CH every 40 min (≈75 g·h^−1^) with 500 ml of fluid-maintained GLUv >90 mg/dl and prevented body mass losses >2%. From a physiological perspective, maintaining glycemia above this threshold likely ensured a continuous supply of exogenous glucose to the working muscles, reducing reliance on hepatic glycogenolysis and delaying the onset of peripheral fatigue associated with glycogen depletion. This finding supports the concept that exogenous CHO availability plays a key role in preserving metabolic homeostasis during repeated high-intensity endurance bouts. Adding a gel containing 45 g of CH during recovery (total ~195 g) promotes rapid glycogen resynthesis, especially when the time between tests is short (< 4 h). This strategy is particularly relevant given that glycogen resynthesis rates are highest immediately post-exercise, when insulin sensitivity and GLU-4 translocation remain elevated, facilitating efficient glucose uptake and storage in skeletal muscle. Therefore, the timing and dose used in this protocol provide experimental support for optimizing recovery kinetics in intermittent endurance context. The combination of low- and high-molecular-weight glucose polymers optimizes absorption and gastrointestinal tolerance without compromising hydration. This combination may enhance intestinal CHO transport capacity by maintaining favorable osmotic gradients and gastric emptying rates, thereby supporting sustained exogenous CHO oxidation and minimizing gastrointestinal limitations, which are a key determinate of endurance performance.

Maintaining stable GLUv and good hydration prevents central and peripheral fatigue and can be integrated into competition simulation training. Specifically, stable glycemia contributes to maintaining central nervous system function and motor drive, while adequate hydration preserves plasma volume, cardiovascular stability, and thermoregulatory efficiency, all of which are critical determinants of endurance performance and fatigue resistance. These findings provide mechanistic evidence supporting integrated CHO fluid strategies to optimize both metabolic and cardiovascular responses during intermittent endurance exercise. As this is a pilot study with a small sample size, the recommendations are tentative and should be adapted to each athlete's intestinal absorption, sweat rate, and body mass. Nutritionists can use these doses as an initial reference and adjust them according to individual characteristics. These results also contribute to refining current CHO intake recommendations by providing specific experimental evidence in a mixed intermittent endurance model, a format that has been less extensively studied compared a continuous endurance exercise. In summary, the strategy of ≈195 g of CH and 1,500 ml of fluids was effective in maintaining GLUv and hydration during repeated efforts, providing a practical basis for endurance sports with short breaks and effort times like those in our protocol. These findings extend current theoretical models of CHO metabolism during exercise by demonstrating that maintaining exogenous CHO availability during both exercise and short recovery periods is essential to preserve metabolic stability and optimize recovery in intermittent endurance formats.

From an applied perspective, these findings can help trainers and physical preparators plan nutrition and hydration strategies during training sessions that mimic competition, allowing athletes to test the specific diet for the race, recovery times, and gastrointestinal tolerance under controlled conditions of high intensity intermittent. Furthermore, the present results provide a framework for periodizing CHO intake not only to support performance but also to enhance recovery efficiency between repeated bouts, thereby contributing to improved training quality, reduced cumulative fatigue, and potentially better lont-term physiological adaptations.

## 5 Conclusions

Within the framework of this pilot and descriptive study, the proposed fluid and carbohydrate (CH) intake strategy was well tolerated and was associated with the maintenance of glycemic levels and body mass within the observed ranges during the evaluated protocol. Exercise at 90% of HRmax produced similar concentrations of GLUv and GLUc in both exercise sectors, consistent with the intake of 50 g of CH per sector. Counterregulatory hormones (NA and GG) showed higher plasma concentrations during the second exercise sector, even in the presence of high CH availability.

Overall, these findings suggest that the tested hydration and CH protocol was tolerable under the simulated relay race conditions and elicited reproducible metabolic and hormonal responses. However, given the small sample size and descriptive nature of the study, these results should be interpreted with caution. Confirmatory studies with larger cohorts and additional physiological markers are needed to validate the current results and optimize nutritional strategies for high-performance endurance events.

## Data Availability

The raw data supporting the conclusions of this article will be made available by the corresponding author upon request.
