# Supplementary material for: An exploratory study of fluid and carbohydrate intake and related metabolic and hormonal responses during intermittent mixed racewalking: implications for the Olympic mixed racewalking relay
Source: Front Nutr. 2026 Jul 3;13:1798548. doi: 10.3389/fnut.2026.1798548 (PMC13376323; doi:10.3389/fnut.2026.1798548)
Supplement: Supplementary file 1 [file Supplementary_file_1.docx]

**Figure 1.** A) Kinetics of venous and capillary glucose concentrations during the exercise protocol. B) Changes in plasma (GLUv) and capillary (GLUc) glucose and insulin (IN) concentrations during the exercise protocol. C) Area under the curve (AUC) of venous and capillary glucose and insulin concentrations. D) Differences in AUC between plasma (GLUv) and capillary (GLUc) glucose. E) Changes in GLUv and cortisol (COR) concentrations during the exercise protocol. F) Changes in GLUv and IN concentrations. Data presented as averages of the five participants.





**Figure 2.** A) Kinetics of plasma concentrations of adrenaline (AD) and noradrenaline (NA) during the exercise protocol. B) Changes in plasma concentrations of NA and cortisol (COR) during the exercise protocol. C) Changes in plasma concentrations of glucagon (GG) during the exercise protocol. D) Changes in plasma concentrations of insulin (IN), COR, plasma glucose (GLUv) and GG during the exercise protocol. E) Changes in plasma concentrations of NA and dopamine (DP) during the exercise protocol. F) Changes in plasma concentrations in plasma glucose (GLUv) and DP. Data presented as averages of the five participants.





**Figure 3.** A) Kinetics of capillary blood lactate concentrations (La^−^) during the exercise protocol. B) Changes in plasma concentrations of La^−^ and free fatty acids (FFA) during the exercise protocol. C) Changes in body mass (BM) during the exercise protocol. D) Changes in plasma glucose (GLUv), La^−^ concentrations and BM during the exercise protocol. E) Changes in rate perceived exertion (RPE) scores during the exercise protocol. F) Changes in plasma concentrations in La^−^ and RPE scores. Data presented as averages of the five participants.





Figure 4. A) Changes in heart rate (HR) during the exercise protocol. B) Changes in plasma concentrations of plasma glucose (GLUv), lactate (La−) and HR during the exercise protocol. Data presented as averages of the five participants.
